# Face masks suitable for preventing COVID-19 and pollen allergy. A study in the exposure chamber

**DOI:** 10.1007/s40629-021-00180-8

**Published:** 2021-07-14

**Authors:** Karl-Christian Bergmann, Sebastian Kugler, Torsten Zuberbier, Sylvia Becker

**Affiliations:** 1 Allergie-Centrum-Charité, Department of Dermatology, Venereology and Allergy, Charité—Universitätsmedizin Berlin, Corporate Member of Freie Universität Berlin, Humboldt-Universität zu Berlin, and Berlin Institute of Health, Berlin, Germany; 2 ECARF (European Centre for Allergy Research Foundation), Berlin, Germany

**Keywords:** Pollen, Allergic rhinoconjunctivitis, Protection to pollen, Pollen allergy, SARS-CoV-2

## Abstract

**Background:**

Since the outbreak of the coronavirus pandemic, the population in Germany has been asked to wear face masks in public areas. The masks are accepted by the public. People with a pollen allergy have an interest in knowing whether masks can also provide protection against pollen and thus prevent symptoms even without medication.

**Method:**

In order to evaluate the potential ‘antipollen effect’ of face masks, 14 adults with confirmed grass pollen-induced allergic rhinoconjunctivitis were exposed to grass pollen for a period of two hours following a standardised protocol. The test was conducted outside of the grass pollen season. The subjects wore either no mask, a medical mask or a FFP2 mask.

**Results:**

Subjects wearing either mask were clearly able to avoid both nasal and conjunctival symptoms. There were no significant differences between the two masks in terms of effect. Mask wearing to prevent pollen exposure clearly supports overall well-being.

**Conclusion:**

Wearing a mask during pollen season can be recommended as an effective nondrug option for people with a pollen allergy.

**Supplementary Information:**

The online version of this article (10.1007/s40629-021-00180-8) contains supplementary material, which is available to authorized users.

## Introduction

As a consequence of the COVID-19 pandemic, the wearing of face masks is now required even in countries such as Germany and is accepted by the majority of the population as a reasonable measure for preventing infection. Unlike countries such as China, face masks are not traditionally worn in Europe, since the air quality is relatively normal. There is therefore no data or any reliable information on the possible influence of masks on the symptoms of people with a pollen allergy.

The situation has changed drastically; masks are also worn by many of the 11 million adults in Germany with a pollen allergy, most of whom suffer from allergic rhinoconjunctivitis [1].

Certain individuals have reported subjectively that they are experiencing fewer symptoms, such as runny nose or itchy eyes, as a result of wearing the mask; however, it remains unclear whether the decrease in symptoms is due to the mask, a lower pollen count in the immediate environment of these individuals or the use of allergy medication.

A survey among nurses in an Israeli hospital revealed that those with allergic rhinitis who wore a medical mask and/or N95 mask (equivalent to a FFP2 mask) while working in the hospital experienced an overall reduction in their allergy symptoms [2]. It can be assumed that pollen, mould spores, animal hair and house dust mite allergens are only present at low levels inside the hospital.

The fact that both medical masks and FFP2 masks offer hospital staff ‘antiviral protection’ of approximately the same magnitude was shown in an overview [8].

In the study presented here, we investigated—for the first time, according to our knowledge—the potential value of mask wearing for people with pollen-induced allergic rhinoconjunctivitis, to a certain degree as a positive collateral benefit in addition to preventing coronavirus infection.

## Method

Subjects: The study included 14 nonsmoking subjects (6 women and 8 men with a median age of 37 years, range 24–63 years) who had been suffering from grass pollen-induced allergic rhinoconjunctivitis for at least two years. In a prick test, the subjects developed a wheal measuring ≥ 3 mm in diameter (grass mix, 30 HEP/ml; LETI Pharma GmbH, 85737 Ismaning, Germany), and they achieved a Total Symptom Score of at least 6 points in previous provocation tests with grass pollen in the allergen exposure chamber (AEC). None of the subjects was undergoing medical treatment or using inhaled medication for bronchial asthma.

Furthermore, none of the subjects had received allergen-specific immunotherapy in the last 5 years; none of them used systemic or local antiallergic drugs for at least 7 days before the first (V1) and until the last exposure (V5) in the chamber.

The subjects were informed about the study verbally and in writing. Approval for this study was based on a positive vote obtained from the ethics commission at Charité (Ethics Commission, Charité Campus Mitte, no. EA1/406/20).

Exposure in the allergen exposure chamber (AEC): The study was carried out in the exposure chamber of ECARF from 16 March 2021 to 15 April 2021. The methodology [4] and evaluation [3] of the study are described.

Exposure procedure: The exposure tests were conducted with grass pollen (grass pollen raw material, 38 ± 2 µm, Phleum pratense; Allergon HB, Angelhom, Sweden) with 4000 pollen/m^3^ according to the preliminary tests for the validation of the AEC. All tests are performed at 21 °C and 55% relative humidity. Before starting the test, the subjects are acclimatised for 20 min without any exposure. The subsequent exposure period for the subjects was 120 min.

The subjects were seated in the AEC according to a random number and retained the same seat for all exposure phases.

On V0, the subjects wore no face masks; on V3, they wore a FFP2 mask (particle filtering half mask, manufacturer: Shengquan, CE2834, EN: EN149:2001 + A1:2009) and on V5 a medical (surgical) mask (manufacturer: MERSUII GB: GB/T 32610-2016).

The intervals between the three exposure phases were seven days for all of the subjects.

Recording of symptoms: The subjects used tablets to document 13 symptoms every 10 min: four eye symptoms (itching, foreign body sensation, lacrimation, redness), four nasal symptoms (itching, sneezing, runny nose, stuffy nose), three bronchial symptoms (wheezing, coughing, shortness of breath) and other symptoms (itchy skin and itchy palate).

The severity of each symptom is rated on a scale of 0 to 3 as follows: 0 = no symptoms, 1 = mild symptoms (clearly present but only very mild, causing a minimal degree of discomfort or none at all), 2 = moderate symptoms (definite presence of symptoms that cause discomfort but are still tolerable) and 3 = severe symptoms (symptom is difficult to tolerate, interferes with the subject’s daily life).

The symptom severity scores are added together to obtain the Total Eye Symptom Score (TESS), Total Nasal Symptom Score (TNSS), Total Bronchial Symptom Score (TBSS), and Total Other Symptom Score (TOSS). The Total Symptom Score (TSS) was calculated as the sum of TESS, TNSS, TBSS and TOSS with a maximum of 39 points.

### Measurements (according to SOPs in the AEC)

Spirometry using EasyOne™ Spirometer (ndd, Medizintechnik AG, Zurich, Switzerland) before V1 and after V5.

Peak flow values using peak flow meters (PersonalBest, Philips GmbH, Herrsching, Germany) before exposure on V1, V3 and V5 and after 30, 60, 90 and 120 min inside the AEC. For all measurements, the highest value of two measurements each was documented.

Peak nasal inspiratory flow (PNIF, Clement Clarke International Ltd., Harlow, Essex, United Kingdom): The PNIF was determined prior to exposure on V1, V3 and V5 and after 30, 60, 90 and 120 min inside the AEC. For all measurements, the highest value of two measurements each was documented.

Nasal secretion: Each subject was given a resealable bag with a packet of tissues for the duration of the exposure. To determine the amount of nasal discharge, the bags are weighed before and after exposure and the weight is documented.

Overall well-being (VAS): The subjects rated their general well-being before, every 30 min during and after allergen exposure in the AEC on a visual analogue scale of 10 cm, which represents the severity level from 0 cm ‘very good’ to 10 cm ‘very poor.’

Follow-up calls (safety calls): Around 24 h after each exposure, the subjects were called as a safety measure; they were asked whether they had any symptoms of a late reaction to the allergen exposure, and any findings were documented. The subjects were asked: “From the moment you left the exposure chamber yesterday until now, have you experienced any allergy or other symptoms?”

Data entry and data protection: The participants’ data were anonymised and entered into a database. All data are managed in accordance with the Berlin Data Protection Act (BlnDSG) and the EU General Data Protection Regulation (GDPR).

Statistical evaluation: The data are evaluated according to protocol including all subjects who started the first exposure and were shown to react to exposure to grass pollen (TSS of ≥ 6). The primary endpoint was the change in TSS, TNSS, TESS and TBSS values over 120 min during exposure. These values were then subjected to a two-sample t‑test on dependent samples (V1/V3, V1/V5 and V3/V5). In addition, all parameters were also evaluated in a descriptive analysis.

COVID-19 hygiene measures: Compliance with a comprehensive set of hygiene and safety measures was ensured throughout the study, from the start of the appointment scheduling until the end of the study. These included patient information, disinfection of all surfaces, writing instruments and medical devices, compulsory mask wearing, gloves and physical distancing in the AEC.

Before beginning the study, the subjects and study staff (study nurse/study doctor) were required to complete a questionnaire about COVID-19 and indicate any symptoms they may have had.

## Results

**Fig. 1:**
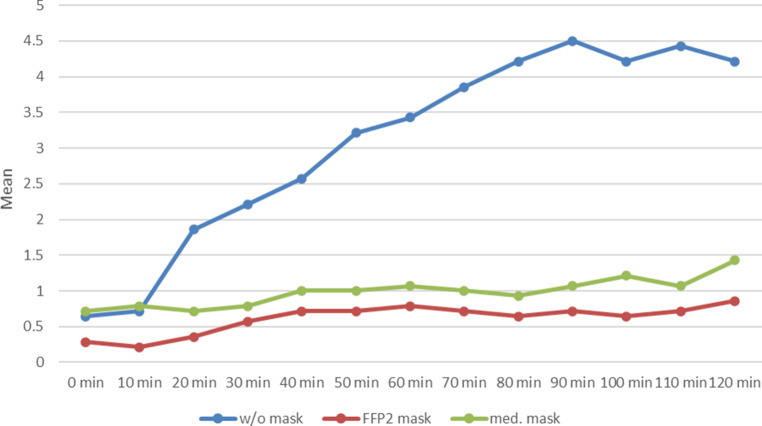
Progression of the Total Nasal Symptom Score (TNSS) over 120 min during provocation with grass pollen without (w/o) mask (*blue*), with FFP2 mask (*red*) and with medical (med.) mask (*green*)

After only a few minutes of exposure, subjects without masks had distinct nasal symptoms in the form of itching, sneezing, rhinorrhoea and swelling of the nasal mucosa, as shown in the Total Nasal Score (Fig. 1). The symptoms reached a plateau after approximately 60–80 min.

**Fig. 2:**
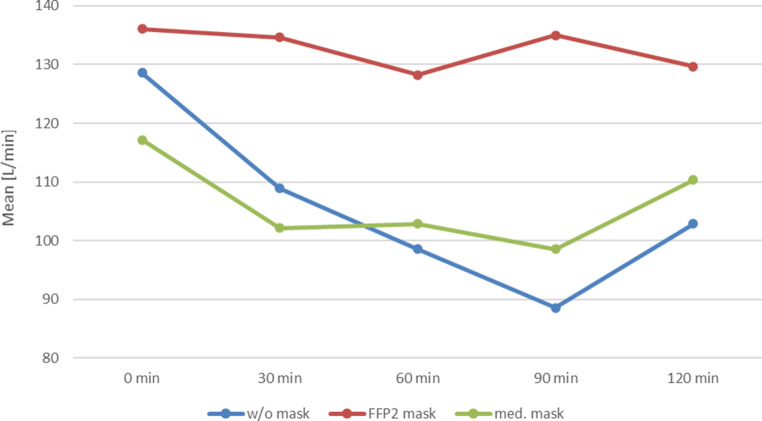
Progression of nasal flow (PNIF) over 120 min during provocation with grass pollen without (w/o) mask (*blue*), with FFP2 mask (*red*) and with medical (med.) mask (*green*)

**Fig. 3:**
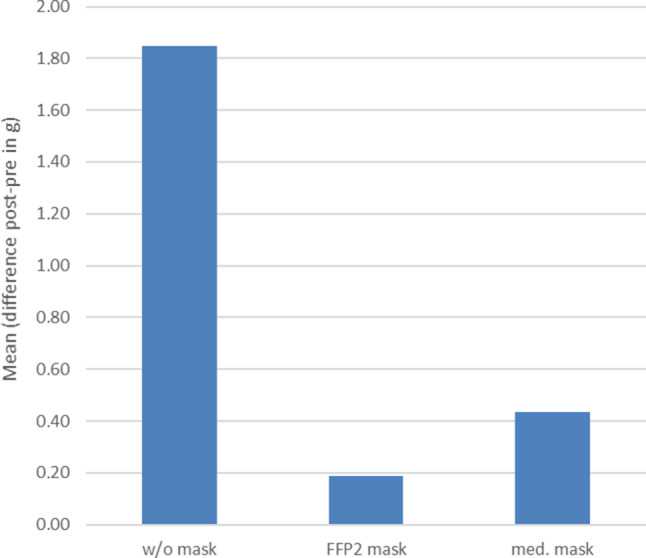
Rhinorrhoea (nasal secretion) over 120 min during provocation with grass pollen without (w/o) mask, with FFP2 mask and with medical (med.) mask

The swollen nasal mucosa led to a significant decrease in nasal flow (Fig. 2) and severe rhinorrhoea (Fig. 3).

Subjects wearing either mask experienced significantly less nasal discomfort, which was evident both in the TNSS, the lower decrease in PNIF and the nearly complete lack of rhinorrhoea (Figs. 1, 2 and 3).

The FFP2 mask was shown to be only slightly more effective than the medical mask in the nasal flow evaluation, and rhinorrhoea was prevented to a greater extent.

**Fig. 4:**
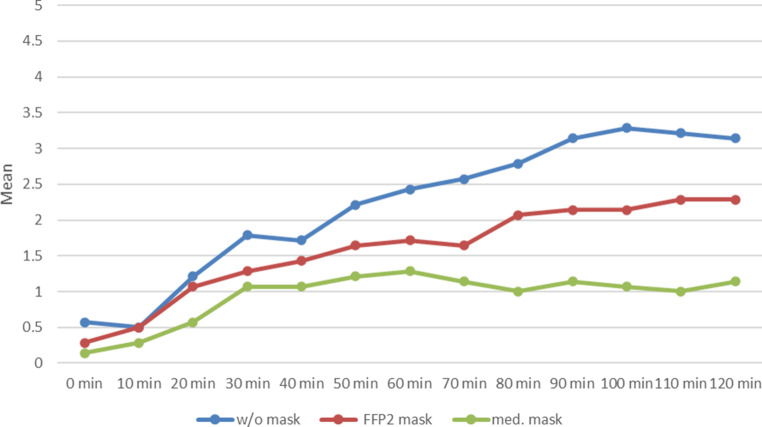
Progression of the Total Eye Symptom Score (TESS) over 120 min during provocation with grass pollen without (w/o) mask (*blue*), with FFP2 mask (*red*) and with medical (med.) mask (*green*)

Both masks also demonstrated a partially protective effect on the eyes; itching of the eyes and lacrimation were less severe. Again, this effect was slightly stronger with the FFP2 mask (Fig. 4) than with the medical mask.

The bronchial symptoms remained negligible and did not exceed severity level 1 in any of the subjects.

The TOSS values were also slightly improved by wearing the masks.

**Fig. 5:**
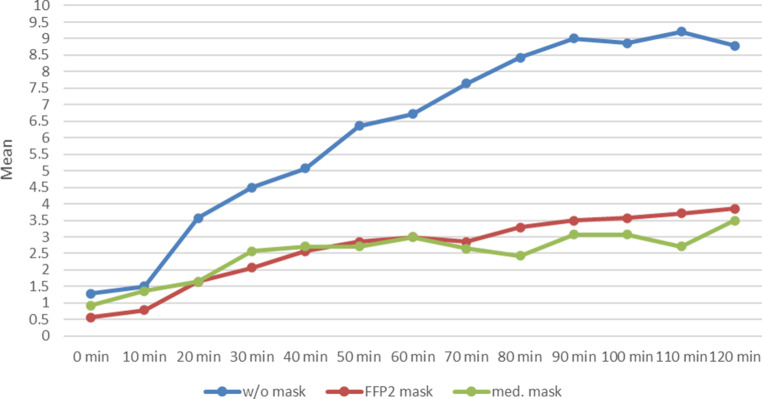
Progression of the Total Symptom Score (TSS) over 120 min during provocation with grass pollen without (w/o) mask (*blue*), with FFP2 mask (*red*) and with medical (med.) mask (*green*)

The overall effectiveness of the masks is revealed when all symptom scores TNSS, TESS, TBSS and TOSS are added together to obtain the Total Symptom Score (Fig. 5).

**Fig. 6:**
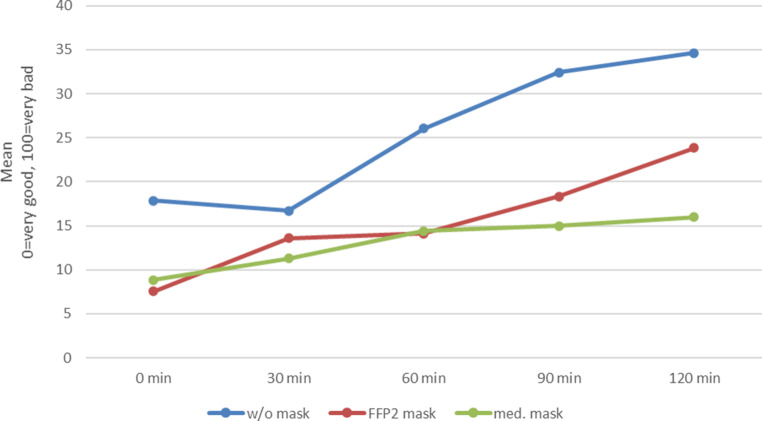
Progression of overall well-being over 120 min during provocation with grass pollen without (w/o) mask (*blue*), with FFP2 mask (*red*) and with medical (med.) mask (*green*)

Overall well-being is supported by the use of either mask (Fig. 6).

All subjects were contacted 24 h after exposure and asked about their well-being. No late reactions were reported. No other abnormalities were reported in terms of safety.

In a hypothesis test using a two-sample t‑test (dependent samples), reliable differences were revealed between symptom severity levels in the TSS, TNSS and TESS when comparing exposure without a mask to the FFP2 mask (V3) and to the medical mask (V5). In a direct comparison of the effect of both mask types for the TNSS and TESS values, there are statistically significant differences; however, these were in an area that is not considered clinically relevant (summary of the statistics in the Table 1).

**Table 1:** Summary of hypothesis tests on the effect of masks compared to exposure without masks and with each other

–	**TSS: none/FFP2**	**TSS: none/med. mask**	**TSS: FFP2/med. mask**
*p*-value	0.00001	0.00005	0.28991
–	**TNSS: none/FFP2**	**TNSS: none/med. mask**	**TNSS: FFP2/med. mask**
*p*-value	0.00001	0.00004	0.00000
–	**TESS: none/FFP2**	**TESS: none/med. mask**	**TESS: FFP2/med. mask**
*p*-value	0.00004	0.00004	0.00010

*med. mask* medical mask, *TSS* Total Symptom Score, *TNSS* Total Nasal Symptom Score, *TESS* Total Eye Symptom Score

## Discussion

There are approximately 11 million pollen allergy sufferers in Germany, many of whom are interested in nondrug options for preventing or at least alleviating their symptoms—in many cases severe—during the respective pollen season. But few options are available whose efficacy is at least partially supported by clinical evidence.

In this study, we investigated—for the first time, according to our knowledge—the potential value of mask wearing for people with pollen-induced allergic rhinoconjunctivitis, to a certain degree as a positive collateral benefit in addition to preventing coronavirus infection. Masks are worn outdoors where airborne pollen is present, and people with allergies have reported feeling less discomfort. However, it remains unclear whether the decrease in symptoms was due to mask wearing or lower pollen levels in their immediate environment.

We used the standardised conditions of an exposure chamber to determine the effect of the most commonly worn medical masks and FFP2 masks in adults suffering from confirmed grass pollen-induced allergic rhinoconjunctivitis for at least two years. The data indicate a strong benefit from both mask types in preventing allergy symptoms in the nose and eyes. There were statistical differences between the two types of masks tested, but these differences were inconsistent and minor, and we do not consider them clinically relevant. This means that both types of masks can be recommended for pollen allergies.

Like medical masks, FFP2 masks are manufactured using multiple layers of filter fleece; their filtering properties are standardised and the manufacturers are required to comply with these standards. The masks serve to protect the wearer and others during the coronavirus pandemic, provided that they do not have a valve.

Medical masks filter particles larger than 3 µm [5], while FFP2 masks block particles up to 0.004 µm. Both mask types are therefore suitable not only for preventing the transmission of coronaviruses [8, 9] but also for filtering pollen types larger than 5 µm in size. Medical masks and FFP2 masks both offer hospital staff ‘antiviral protection’ of approximately the same magnitude, which should also be useful for local (eye) or inhaled (nose, bronchial) contact with airborne pollen.

The masks must fit correctly in order to be effective. Trials on hospital staff have shown that correct mask fitting can be practised in short programmes with a demonstrable effect [6]. For this reason, fit tests, such as those performed with bitter solutions, are considered important for healthcare workers and are strongly recommended [7]. In our study, no instruction was given and no fit tests were carried out on the masks so that their effect could be investigated under real-world conditions. Please note, however, that pollen allergy sufferers who want to use masks to prevent symptoms should ensure that their masks fit properly in order to increase their effectiveness if necessary.

In this study, the subjects were exposed to a high level of grass pollen (4000 pollen/m^3^) over a period of two hours; as evaluated in allergy sufferers, this safely triggers allergy symptoms and leads to a plateau [3]. Higher or longer exposure does not lead to any further worsening of the symptoms. This intensity of exposure can therefore be used to obtain robust symptom data, which were agreed with the test subjects in advance.

The study was conducted outside of the grass pollen season in Berlin in order to rule out the influence of environmental airborne pollen during the study. The low levels of symptom severity on V0, including the data on overall well-being, demonstrate that no previous exposure to other allergens can be assumed to have influenced the course of symptoms that occurred as a result of exposure to grass pollen. The influence of medication was completely excluded by discontinuing all medications for at least 7 days before beginning the study and throughout the study period.

### Strengths and weaknesses of the study

The study included only 14 subjects, which is a relatively small number of participants and significantly lower than the planned number of 30 subjects. This was due to the pandemic, which prevented many subjects from accepting our invitation to the study, despite the comprehensive hygiene and safety measures in place. This was also the case with other studies [10] and had to be accepted. Furthermore, we were unable to test a ‘placebo mask’ and are as of yet unaware of the existence of such a mask.

The strength of the study lies in the standardised conditions of the exposure chamber. These conditions made it possible to objectively compare the occurrence of allergy symptoms without any mask and with two different masks; similar data cannot be obtained through simple surveys. The study supports a new recommendation for patients with hay fever that allows them to reduce or completely eliminate their symptoms, especially on days with a high pollen count, using a simple, nondrug approach. Nondrug recommendations are frequently requested by people with allergies.

Could mask wearing also be helpful against other allergic rhinoconjunctivititis triggers, for example, in people allergic to mould spores, cats or mites in certain situations (e.g. a high level of alternaria spores, or when visiting homes with cats)? This may be possible due to the retention capacity of masks such as the FFP2, but still needs to be confirmed in a similar study before such claims can be made.

## Conclusions

The wearing of face masks, such as medical masks or FFP2 masks, provides significant protection against grass pollen in people with pollen-induced allergic rhinitis and significantly decreases allergy symptoms in the nose and eyes. In practice, there is no significant difference between the two mask types in terms of effect—they are equally effective and are both recommended.

The effect is also present at high pollen levels.

Whether pollen-induced asthmatic symptoms can be reduced or excluded has not been proven.

The wearing of face masks is the most intensive nondrug measure for preventing pollen-induced symptoms in allergy sufferers.

## Supplementary Information

Appendix
