# Supplementary material for: Face masks suitable for preventing COVID-19 and pollen allergy. A study in the exposure chamber
Source: Allergo J Int. 2021 Jul 14;30(5):176–82. doi: 10.1007/s40629-021-00180-8 (PMC8278371; doi:10.1007/s40629-021-00180-8)
Supplement: Supplementary file 1 — Appendix [file 40629_2021_180_MOESM1_ESM.pdf]

Electronic appendix

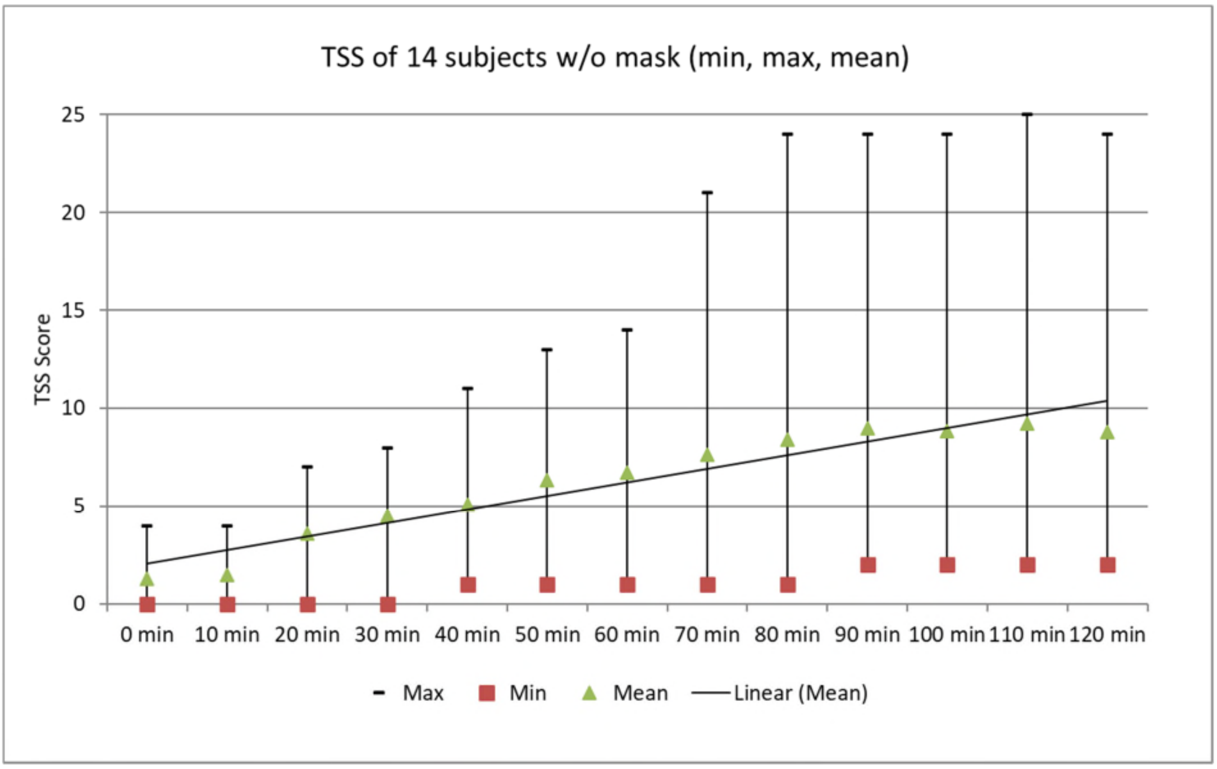

Fig. 1 – Appendix:: Total Symptom Score on V1 (without mask) over 120 minutes with mean, minimum and maximum.

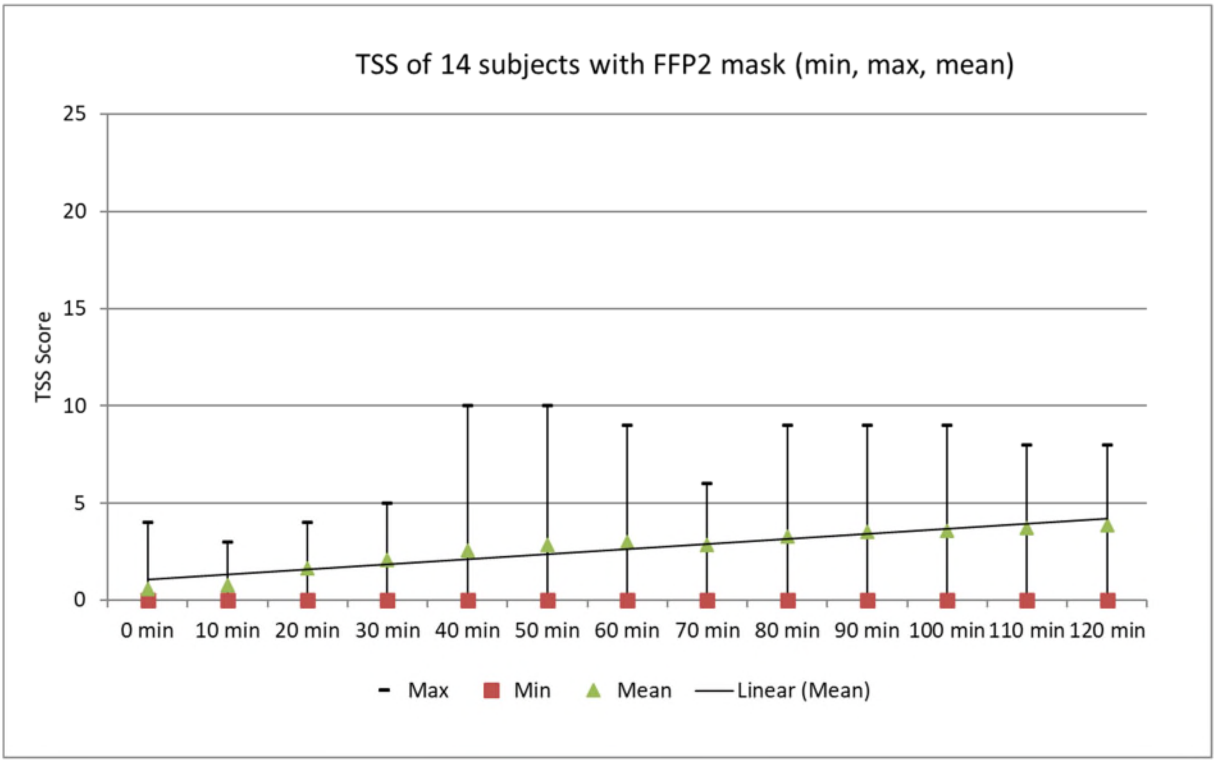

Fig. 2 – Appendix: Total Symptom Score on V3 (with FFP 2 mask) over 120 minutes with mean, minimum and maximum.

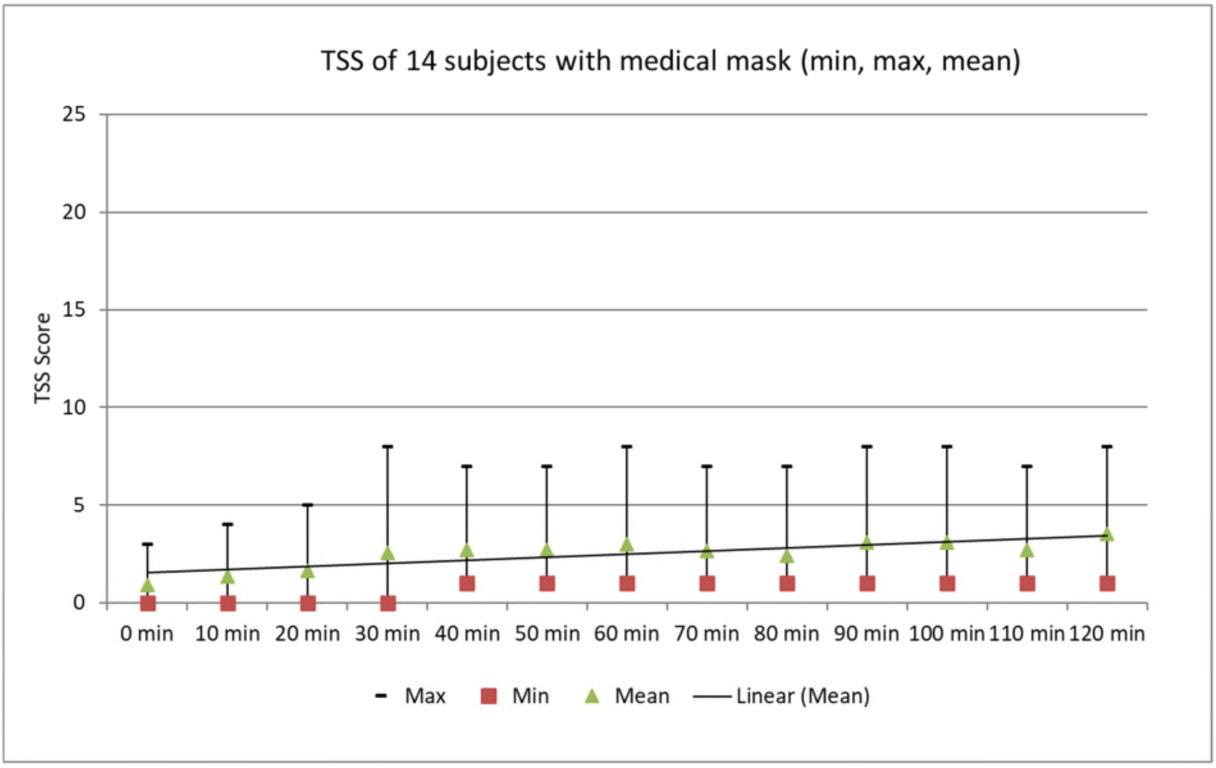

Fig. 3 – Appendix: Total symptom Score on V5 (medical mask) over 120 minutes with mean, minimum and maximum.
